# Dataset on the human body as a signal propagation medium for body coupled communication

**DOI:** 10.1016/j.dib.2023.109892

**Published:** 2023-12-01

**Authors:** Juris Ormanis, Vladislavs Medvedevs, Anastasija Sevcenko, Vladimir Aristov, Valters Abolins, Atis Elsts

**Affiliations:** Institute of Electronics and Computer Science (EDI), Riga LV-1006, Latvia

**Keywords:** Intra-body communication, Signal loss, Body area networks, Wearables

## Abstract

Signal loss models are frequently utilized by wireless communication researchers and engineers to predict received signal strength, optimize system parameters, and conduct feasibility studies. However, novel communication methods such as Body-Coupled Communication (BCC) that are suitable for Body Area Networks formed by wearable devices currently lack readily available signal propagation models. In this data article, we present a galvanic-coupled BCC signal loss and bioimpedance dataset, which serves as a foundation for building such models. This extensive dataset consists of experimental data recorded from 30 volunteer test subjects. The experimental setup involves a tunable signal generator transmitting continuous wave signals, along with two oscilloscopes recording the transmitter-side and receiver-side voltages. From these measurements, we compute the signal loss over the body, and the transmitter-side impedance. The transmitted signal frequencies range from 50 kHz to 20 MHz, with discrete steps. The primary application of this dataset is to enable empirical-data-supported modeling in the human body as a BCC signal propagation medium, which will help to explore how the properties of the human body, the measurement locations, and the signal frequency impact the signal loss.

Specifications Table

**Table:** 

Subject	Engineering
Specific subject area	Telecommunications Engineering
Data format	Raw, Filtered
Type of data	CSV and JSON files, Graph
Data collection	Experimental measurements in a laboratory using a signal generator, oscilloscopes, computing units, and custom measurement equipment on volunteer test subjects.
Data source location	Institute of Electronics and Computer Science (EDI).
Data accessibility	Repository name: Dataset on the Human Body as a Signal Propagation Medium Data identification number: 10.5281/zenodo.8214497 Direct URL to data: https://zenodo.org/record/8214497

## 1 Value of the Data

• The dataset [14] will allow anyone interested in Body Coupled Communication (BCC) to investigate and simulate BCC systems without going through the extensive effort required to set up and validate an experimental measurement system [1], [2], [3], [4], [5], [6].
• The main application is building models of the expected signal loss in the human body, depending on the properties of the body, such as height, weight, body composition and others; the measurement locations; and the signal frequency [7], [8], [9].
• One target group is the designers of Body Area Networks (BAN), who will be able to use the models to determine constraints on their systems such as the minimum required transmission power.
• Another target group is BAN protocol developers and simulator builders, who can integrate the models in their toolchain in order to get a more realistic simulations of their protocol performance [10].

## 2 Data Description

### 2.1 Overview

**Fig. 1:**
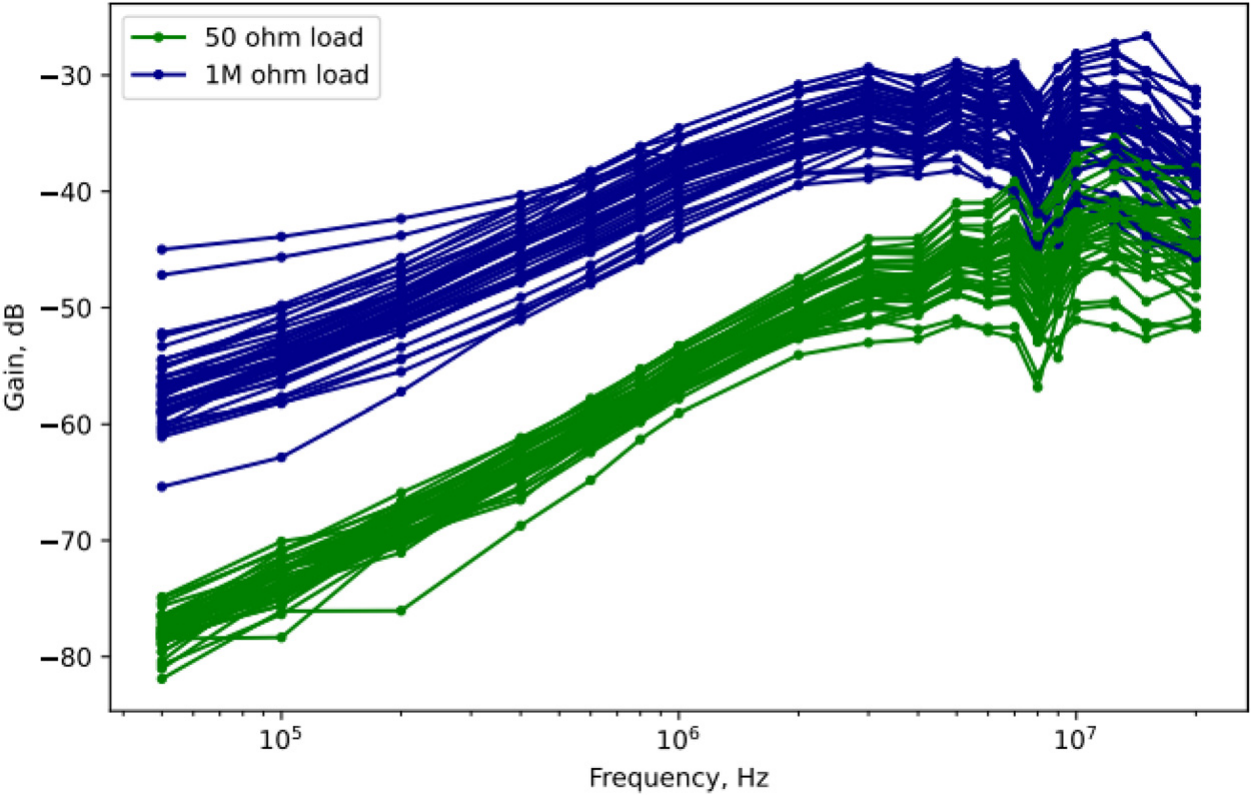
Visualization of a sub-sample (*N* = 50) of the dataset. Signal gain on the receiver plotted against measurement frequency. Each measurement repeated with 50 Ω and 1 mΩ load resistance.

The overview of the dataset is given in Table 1. Fig. 1 visualizes a subset of the dataset. We note that while the existing research on signal loss in the body typically only reports the results obtained using a single, constant load resistance [11], [12], [13], our setup recorded the signal loss at two different settings: at 50 Ω and 1 mΩ load resistance, in the receiver-side measurement unit.

**Table 1:** Dataset overview.

Variable	Value
Number of test subjects	30
No. transmitter locations	6
No. receiver locations	6
No. measurement frequencies	19
Frequency range	50 kHz – 20 MHz
Input voltage V_G_	1 V
Load resistance	50 Ω and 1 mΩ

All data was recorded in measurement sessions during January to June 2023, in a single, specifically fitted room in EDI premises. Several participants had multiple measurement sessions during multiple different days.

**Fig. 2:**
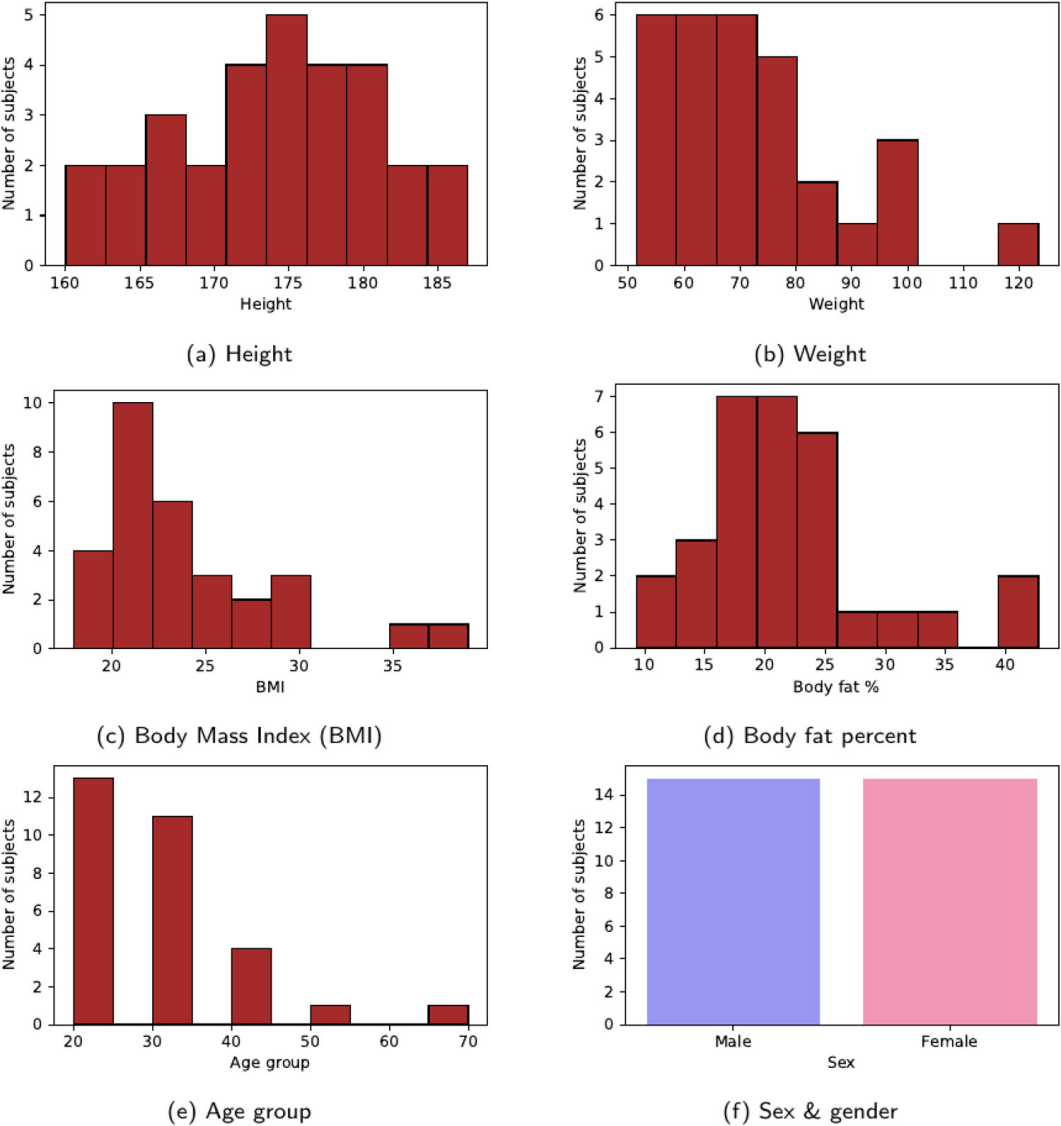
Characteristics of the measurement group.

The overview of the measurement group is given in Table 2 and in Fig. 2.

**Table 2:** Measurement group overview.

Variable	Mean (Std)
Height	174.10 (7.15)
Weight	72.85 (16.26)
BMI	23.94 (4.70)
Body fat%	21.53 (7.55)
Age group	29.0 (11.25)
Male/female ratio	50%

### 2.2 Files in the Dataset

The files in the dataset are structured as follows:

RawData

\- ID1256

\- BCC Measurement Info Sheet ID1256_12.06.23_1_1M_50.xlsx

\- ID1256_12.06.23_1_1M_50.json

\- ID1686

\- BCC Measurement Info Sheet ID1686_04.07.2023_1_1M_50.xlsx

\- BCC Measurement Info Sheet ID1686_15.02.2023_1_1 M.xlsx

\- ID1686_04.07.2023_1_1M_50.json

\- ID1686_15.02.2023_1_1 M.json

…

all_measurements.csv all_measurements_by_freq.csv all_measurements_filtered.csv all_measurements_by_freq_filtered.csv summary_of_subjects.csv

… other files …

The intention of the CSV files is as follows:

• all_measurements.csv – the key measurement results extracted from the JSON files.
• all_measurements_filtered.csv – same, but after z-score filtering to remove outliers more than two standard deviations away from the mean.
• all_measurements_by freq.csv – the key results extracted from the JSON files, single frequency per row.
• all_measurements_by freq filtered.csv – same, but after z-score filtering
• summary_of_subjects.csv – key statistics on the subjects from the experiment information sheets.

### 2.3 CSV File Format

Each CSV file contains multiple columns:

• subject id – participant's random unique ID
• experiment id – experiment's number, in case a single subject had multiple measurement sessions
• height – participant's height, cm
• weight – participant's weight, kg
• BMI – body mass index, computed from the valued above
• body fat% – body composition in terms of fat as% of the total body mass, as estimated by bioimpedance scales
• age group – age rounded to 10 years, e.g. 20, 30, 40 and so on
• male – 1 if the participant is male, 0 if female
• tx point – transmitter point number
• rx point – receiver point number
• distance – distance, in relative units, between the tx and rx points. Not scaled for each participant individually, in terms of participant's height and limb dimensions.
• tx point fat level – transmitter point location's average fat content metric. Not scaled for each participant individually.
• rx point fat level – receiver point location's average fat content metric. Not scaled for each participant individually.
• total fat level – sum of rx and tx fat levels
• bias – constant term to simplify data analytics, always equal to 1.0

The multi-frequency CSV files also contain the following columns:

• tx abs Z…- transmitter-side impedance, as computed by the process json files.py script from the experimental measururements.
• rx gain 50 f…– experimentally measured gain on the receiver, in dB, using 50 Ω load resistance.
• rx gain 1 M f…– experimentally measured gain on the receiver, in dB, using 1 mΩ load resistance.

The single-frequency CSV files contain just these three columns:

• frequency – measurement frequency.
• rx gain 50 – receiver gain using 50 Ω load resistance.
• rx gain 1 M – receiver gain using 1 mΩ load resistance.

### 2.4 JSON File Format

JSON files contain the complete and unfiltered measurement data, as well as noise measurements in each frequency. The JSON file format is as follows: for every pair of points this type of entry is created:

{

// Transmitter:

{

"DeviceType": 1,

// 1 - transmitter, 2 - receiver

"StartTime": "hh:mm:ss",

"TransmitterPoint": 0–33,

"ReceiverPoint": 0–33,

"Frequencies": array[],

// array of frequencies*/,

"Measurements1M": array[array[10]],

// measurements with 1 M load

"Measurements50": array[array[10]],

// measurements with 50ohm load

},

// Receiver:

{

"DeviceType": 2,

// 1 - transmitter, 2 - receiver

"StartTime": "hh:mm:ss",

"TransmitterPoint": 0–33,

// same as TransmitterPoint in Transmitter

"ReceiverPoint": 0–33,

// same as ReceiverPoint in Transmitter

"Frequencies": array[]

// same as Frequencies in Transmitter

"Measurements1M": array[array[10]],

// measurements with 1 M load

"Measurements50": array[array[10]],

/ss/ measurements with 50ohm load

},

…

}

### 2.5 Information Sheet

An experiment information sheet is included for each measurement session. It contains a detailed information about the participant and the environmental conditions during the session:

• Environmental factors:
  ○ Room temperature
  ○ Air humidity
• Participant characteristics:
  ○ Age
  ○ Sex (M/F)
  ○ Height (cm)
  ○ Weight (kg)
  ○ Body fat percent
  ○ Body Mass Index
  ○ Hand-hand distance (cm)
  ○ Medial ankle-groin distance (cm)
  ○ Xiphoid process to medial ankle (cm)
  ○ Skin temperature: forehead
  ○ Skin temperature: wrist
  ○ Sweat on skin (Y/N)
  ○ Complaints about being cold in the room (Y/N)
• Body circumferences:
  ○ LHW1 (Left Hand Wrist) (receiver side)
  ○ LLC (Left Leg Calf) (receiver side)
  ○ LLK (Left Leg Knee) (receiver side)
  ○ LLB (Left Leg Bicep) (receiver side)
  ○ LLQ (Left Leg Quadricep) (receiver side)
  ○ LLAJ (Left Leg Achilles Joint) (receiver side)
  ○ RHW1 (Right Hand Wrist) (transmitter side)
  ○ RHE (Right Hand Elbow) (transmitter side)
  ○ RHT (Right Hand Triceps) (transmitter side)
  ○ RHD (Right Hand Delta) (transmitter side)
  ○ LOIN (transmitter side)
  ○ CHEST (transmitter side)

### 2.6 Other Files

The dataset also contains the full experimental protocol, and example scripts for data processing.

## 3 Experimental Design, Materials and Methods

**Fig. 3:**
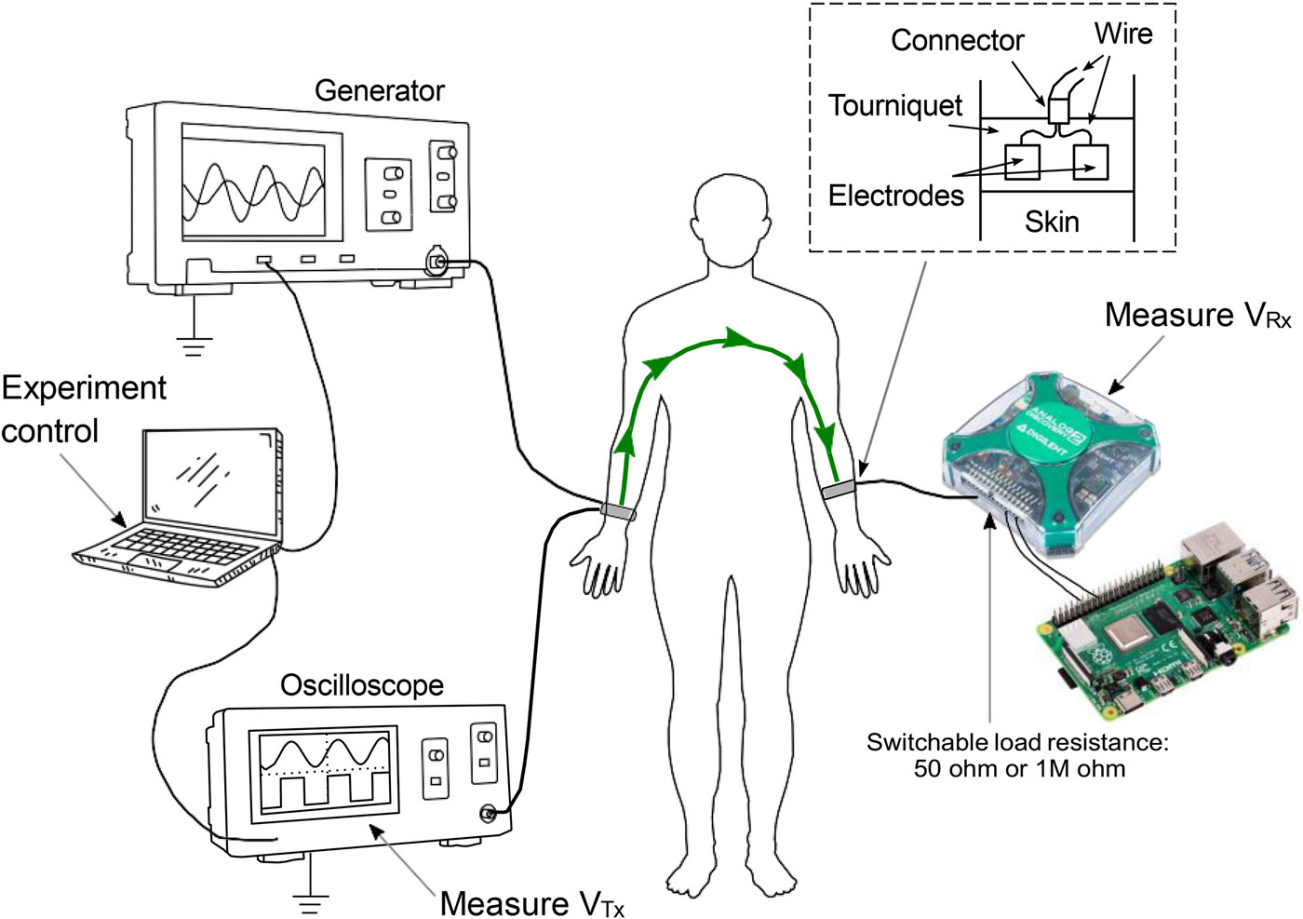
Simplified schematic overview of the setup. Multiplexer, demultipler units, and other electrode pairs not shown.

**Fig. 4:**
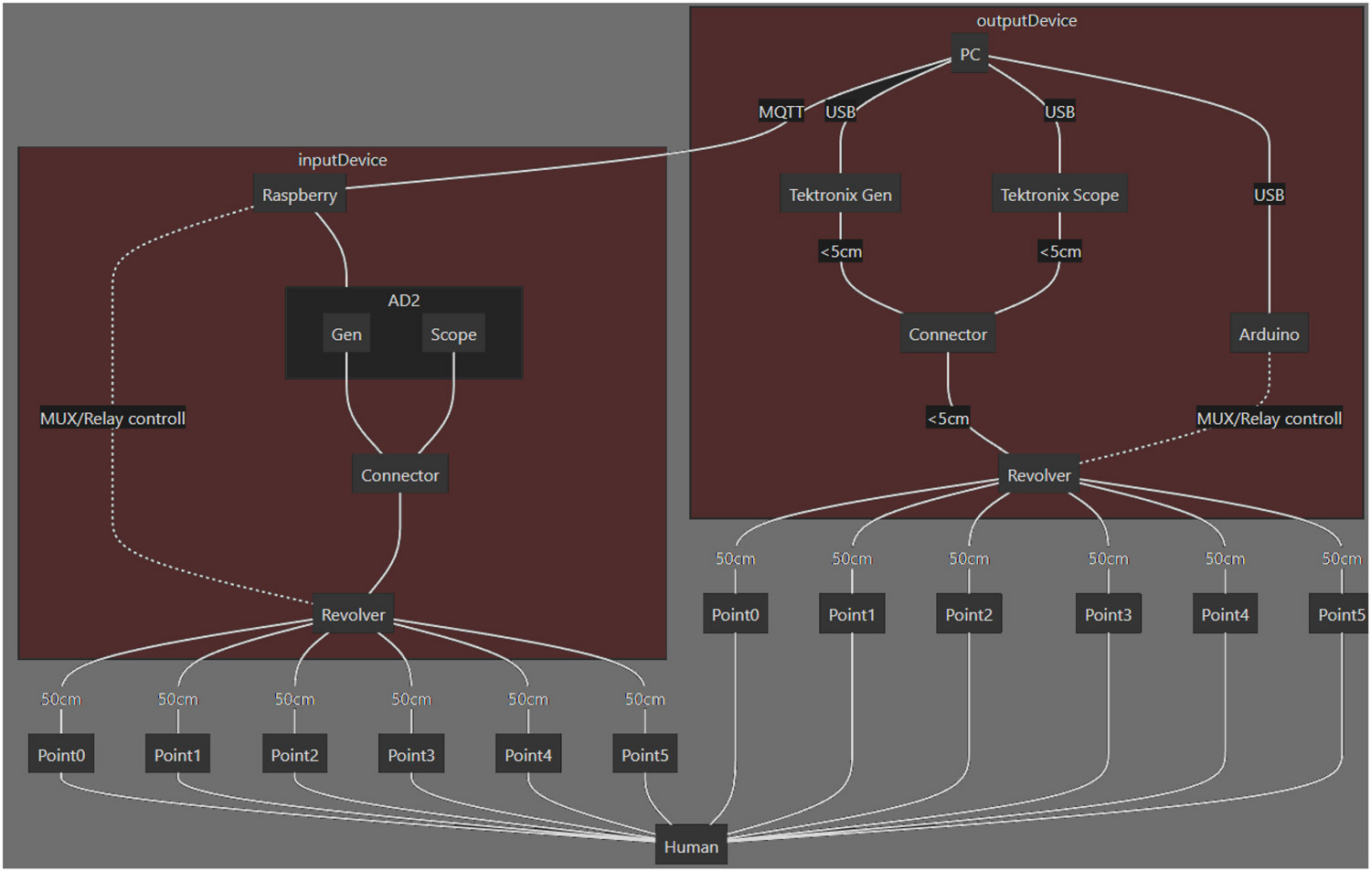
Schematic overview of the setup connections.

The experimental setup (Figs. 3, 4) integrates hardware, software, and procedural elements.

The experiments took place in an unoccupied office room equipped with the couch, the measurement equipment, two chairs for the experimenters, a table and a clothes rack. The target temperature on the room was 20° (±1° deviations permitted); to control that, the room had a hot water radiator connected to the building's central heating system, an electrical radiator for a more fine-grained temperature tuning, and easy-to-open windows. We selected an unoccupied room to ensure privacy guarantees for the participants, and to ensure that there are no changes in the furniture, equipment etc. throughout the data collection phase.

### 3.1 Procedure

**Fig. 5:**
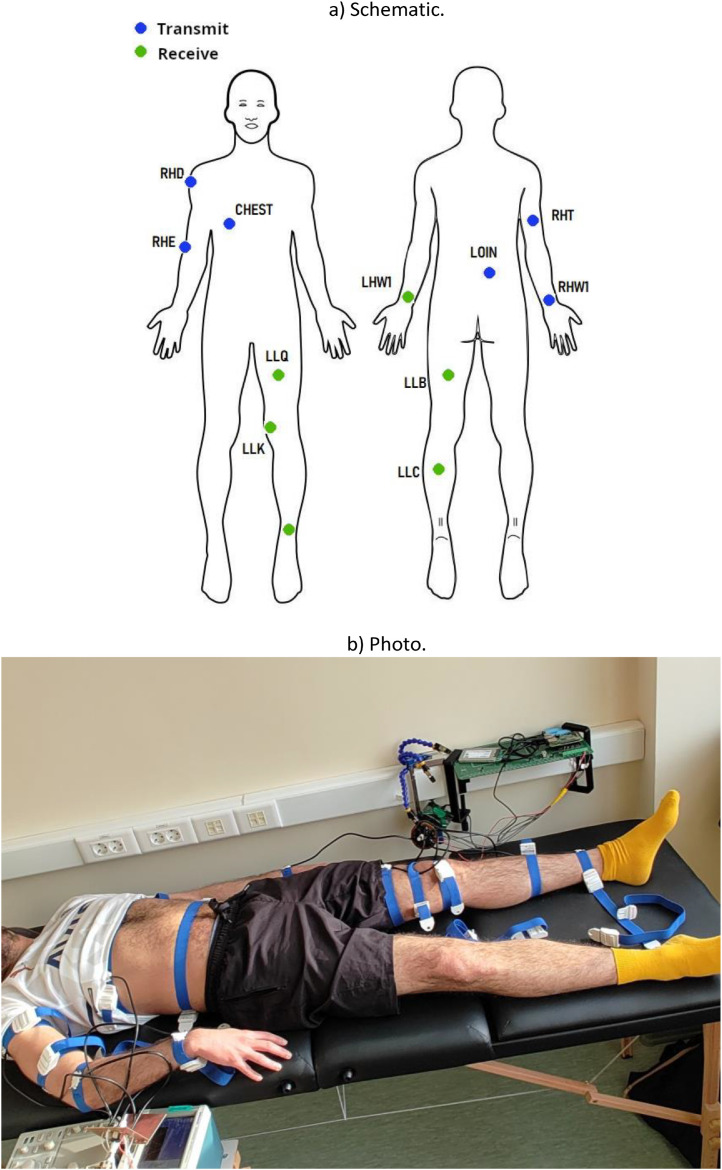
Electrode placement locations.

The full protocol is described in the document “Experimental measurement protocol of the BCC project”, included in the dataset itself. To briefly summarize it, in each measurement session the experimenters measure the signal loss over the experiment subject's body, using six different transmitter locations and six different receiver locations (Fig. 5), for a total of 36 pairs of points. The electrodes are fastened to the measurement locations using elastic tourniquets. A stable skin-to-electrode contact is achieved by applying a liquid ECG gel on test subject's skin below the electrode contact plates. After connecting the 12 pairs of electrodes, the measurement process is automated and performed without operator intervention. During the measurement process the test subject is laying in a supine position on a medical couch. The test subject is instructed to abstain from movements during the process as much as possible.

### 3.2 Hardware

**Fig. 6:**
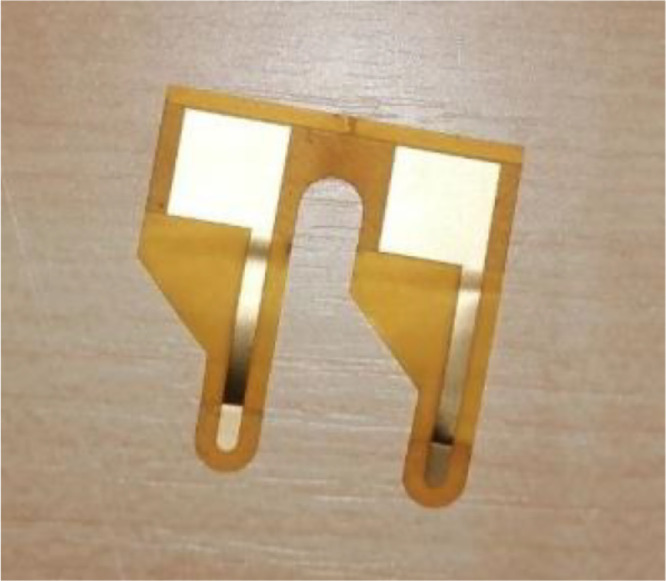
The custom electrodes used in the measurements.

The hardware components in the setup include:

1. Tektronix AFG3252C function generator. Generates the continuous wave signals on the transmit-side electrodes.
2. Tektronix MSO4032 oscilloscope. Measures the voltage *V_Tx_* on the transmit-side electrodes, and allows the operator to visualize and control the process.
3. Analog Discovery 2 (AD2) unit. The unit is configured in the oscilloscope mode, and serves as a signal receiver that measures the voltage *V_Rx_*.
4. Multiplexer and demultiplexer units (Fig. 7). Each unit has a single central and six peripheral connectors, switched via relays, in order to automate measurements at multiple receiver and transmitter locations in a single session. The demultiplexer unit is placed in the path between the signal generator and the transmit-side electrodes. The multiplexer unit is placed between the receive-side electrodes and the AD2 unit.
5. Gold-plated flexible electrodes (Fig. 6). Connected to the test subject body, and used to transmit and receive signals.

**Fig. 7:**
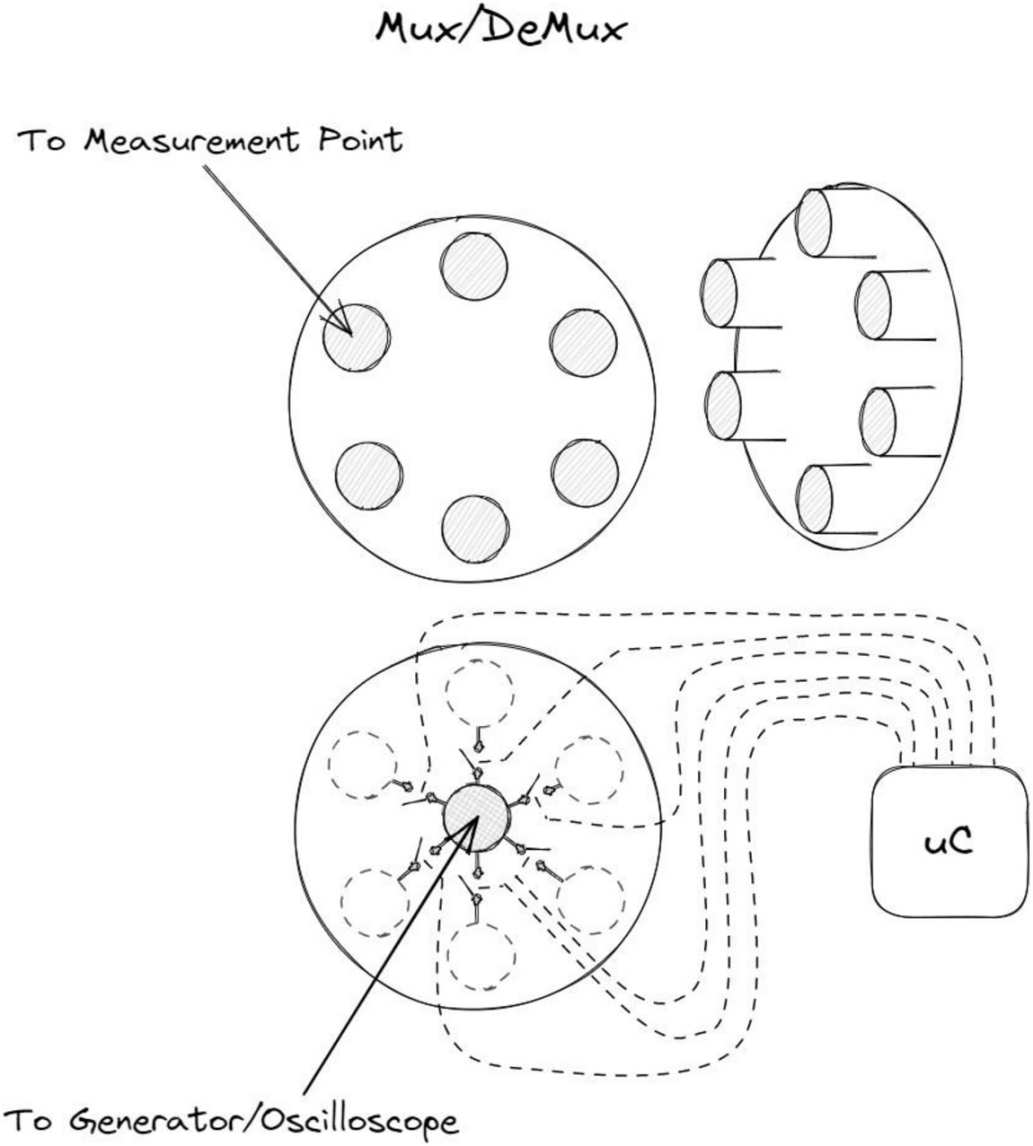
Multiplexer and demultiplexer unit.
6. RG58 coaxial cables. Connect between the other units in the system. The lengths of these cables were maintained under 50 cm, as below this specified distance, wave effects showed negligible disruption to our setup.
7. Raspberry Pi 4 single-board computer. Control the AD2 unit and reads its data.
8. Laptop. Controls the experiment and record the data in JSON files.
9. The treatment couch (Fig. 5). Used for participant to lies down. It consists of a wood frame cushioned by foam and enveloped in synthetic leather. The only metallic constituents of this couch are the bolts reinforcing the wooden joints and the metallic mechanisms responsible for the adjustment of the leg and head positions. This couch resembles the typical design of massage couches.

**Fig. 8:**
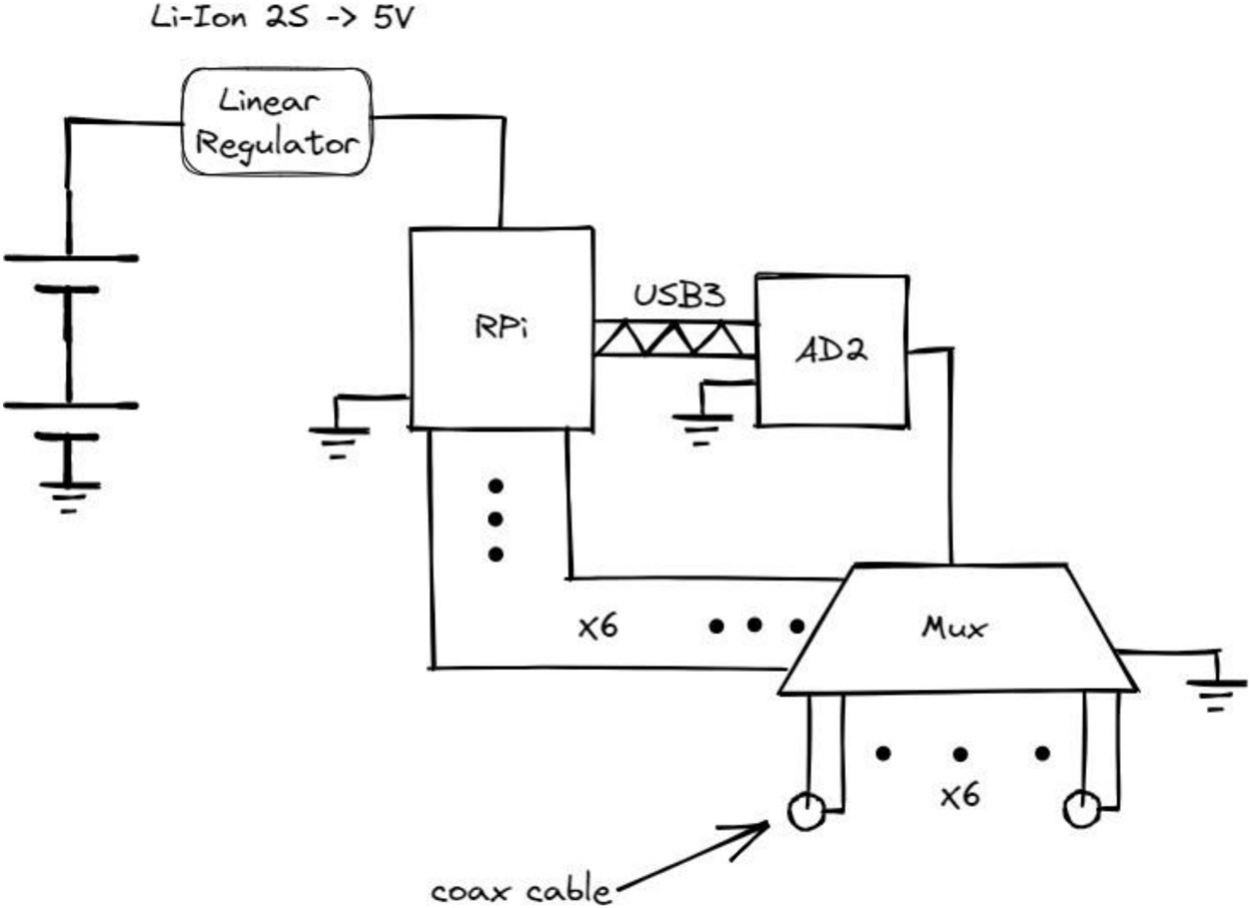
Block diagram of the receiver-side equipment.

The receiver-side equipment (Fig. 8) includes a multiplexer, AD2 unit, and the Raspberry Pi. It is powered from a battery and thus electrically de- coupled from the transmit-side equipment. A WiFi connection is used to communicate between the laptop and the Raspberry Pi.

### 3.3 Software

**Fig. 9:**
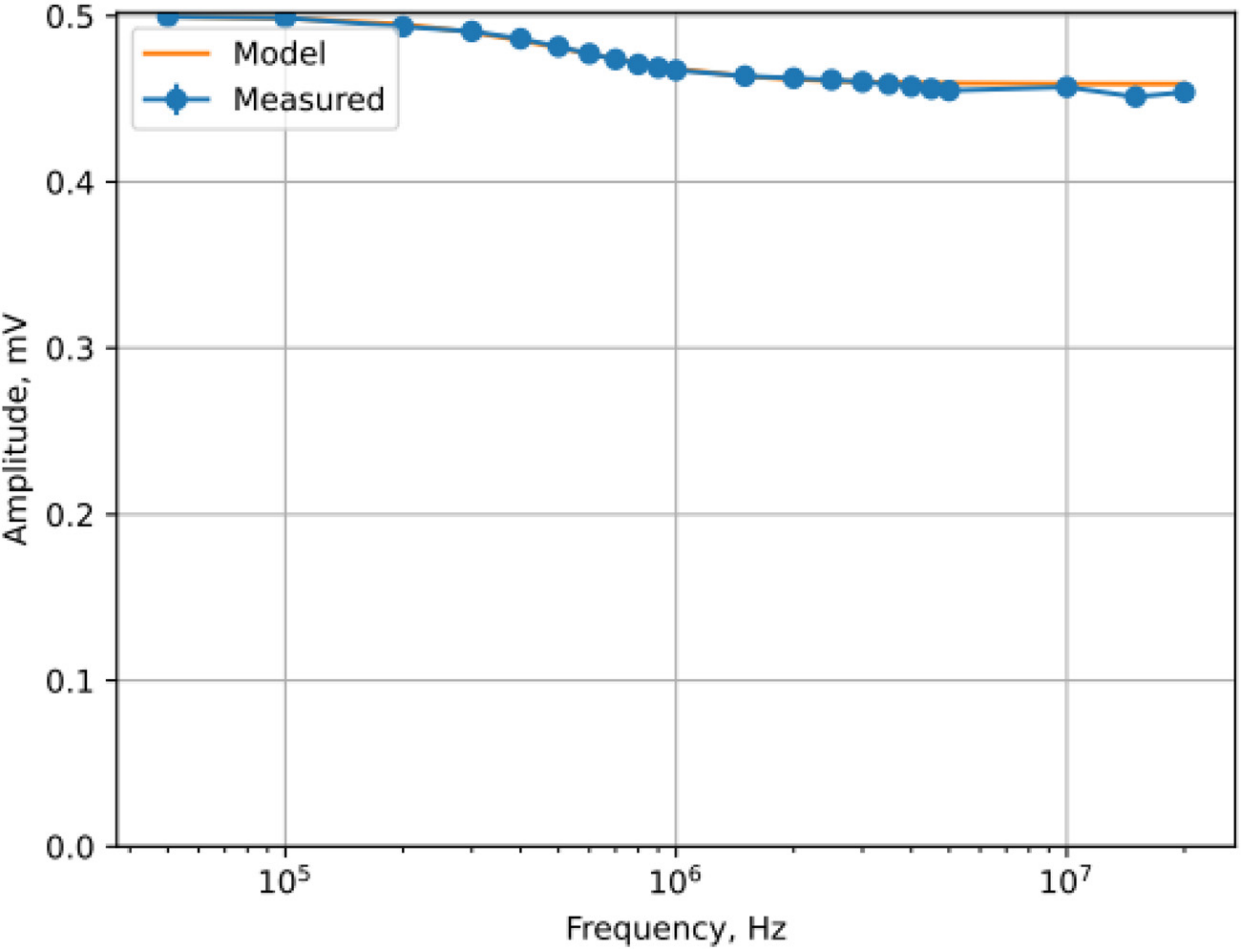
Receiver-side validation: example of a calibration experiment with AD2 unit using a reference load of 280 Ω + 1000 pF impedance.
• Control software. Running on the laptop. Communicates with the other scripts over MQTT. For each frequency in the list, it triggers the transmitter side script to start the signal generation and measurement process. Once the V_Tx_ measurement on the transmitter-side oscilloscope is completed, the control script instructs the receiver-side script to start its measurement. After the receiver-side script completes and acknowledges the measurement, the control script moves on to the next frequency. This iterative process is carried out until all the frequencies listed have been measured. Once the final frequency has been successfully measured, the data is recorded in a JSON file, and the multiplexer and demultiplexer transition to the next pair of electrodes.
• Post-processing software. Computes the transmitter-side impedance and the signal loss from the raw voltage values using the formula:
  $$gain_{Rx}=20\;\times log_{10}\frac{V_{Rx}}{V_{Tx}}$$

• Transmitter-side Python script. Running on the laptop. Sets the desired frequency and voltage *V_G_* on the generator, switches the demultiplexer to connect the generator output with the desired transmitter electrode pair on the participant's body, and records oscilloscope data that measures the voltage drop on that electrode pair.
• Receiver-side Python script. Running on the Raspberry Pi. Uses the multiplexer to select which electrode pair to measure, and triggers the sample collection on the AD2. The amplitude of the signal is recovered using a Fourier-transform based filter as follows: to measure the receiver-side voltage *V_Rx_* at a desired frequency it records a *N* = 8000 sample buffer, computes the Fourier transform *F* on that buffer, and uses the frequency-domain amplitude at the harmonic *h* corresponding to the generator signal's frequency *f*. More formally, using the received signal *S* sampled at rate *R* = 100 million samples per second we compute:
  $$Spectrum=\frac{2}{N}\left|F\left(S\right)\right|$$
  $$h=N\frac{f}{R}$$
  VRx=Spectrum[h]
• Noise resistance and accuracy. The filter described above effectively mitigated any noise during the experiment that was outside our target frequency, including potential 50 Hz noise from the power cables. The processing algorithm and hardware have been validated on reference loads (resistive, capacitive, and combined) and shown to achieve *<*1% error in 4 Mhz and lower frequencies (Fig. 9). For increased stability, it repeats the buffer collection and *V_Rx_* computation process ten times, and reports all ten values to the control script. It also switches the load resistance of the receiver 1 mΩ and 50 Ω, using a custom schematic with a 50 Ω resistor, connected directly to the AD2 unit.

We include example post-processing scripts as well as their outputs in the dataset. The scripts demonstrate how to extract information from the JSON files to a more convenient format, how to remove clearly invalid measurements, and, how to filter out statistical outliers.

### 3.4 Validation

**Fig. 10:**
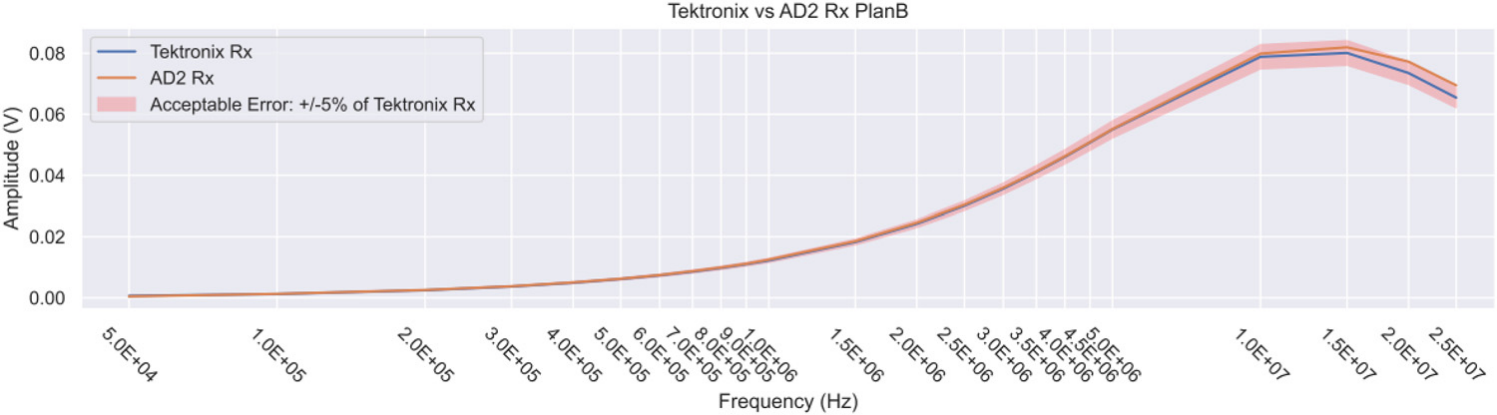
Receiver-side validation: AD2 unit results. The Tektronix oscilloscope is used as a gold standard.
• Testing the electrical connections and signal integrity between the hardware components.
• Testing the functionality of the software components.
• Conducting a pilot study with a small group of participants to evaluate the data collection process and to refine the experimental protocol.

We validated the experimental setup through a series of tests to ensure the accuracy and consistency of the measurements. They included:

• Validation of the generator, oscilloscope, and the AD2 unit to ensure the accuracy of the generated wave forms and measurements (Fig. 10).

## Limitations

Measurements in all measurement frequencies up to and including 20 MHz have been validated with reference resistive, capacitive and mixed loads, and have shown <10% measurement error in the validation. However, due to multiple reasons, including the limited sampling frequency of 100 million samples per second on the receiver-side oscilloscope, the measurement error grows with the frequency.

## Ethics Statement

Informed consent was obtained from the test subjects. The research was carried out in accordance with the Declaration of Helsinki, based on the EDI Ethical committee approval, protocol number Nr. 2–22.

## Data Availability

Dataset on the Human Body as a Signal Propagation Medium (Original data) (Zenodo)
